# Effect of Fique Fibers in the Behavior of a New Biobased Composite from Renewable Mopa-Mopa Resin

**DOI:** 10.3390/polym12071573

**Published:** 2020-07-16

**Authors:** José Herminsul Mina Hernandez, Edward Fernando Toro Perea, Katherine Caicedo Mejía, Claudia Alejandra Meneses Jacobo

**Affiliations:** 1 Grupo Materiales Compuestos, Universidad del Valle, Calle 13 No. 100-00, 76001 Cali, Colombia; kathe.caicedo@hotmail.com (K.C.M.); alejandrameneses91@gmail.com (C.A.M.J.); 2 Grupo Sistemas de Gestión Científica y Tecnológica, Universidad Nacional Abierta y a Distancia, Av. Roosevelt No. 36-60, 110311 Cali, Colombia; edward.toro@unad.edu.co

**Keywords:** Mopa-Mopa resin, biobased composite, fique fibers, wood–plastic

## Abstract

A fully biobased composite was developed using a natural resin from the *Elaeagia Pastoensis* *Mora* plant, known as *Mopa-Mopa* reinforced with fique fibers. Resin extraction was through solvent processing reaching an efficient extraction process of 92% and obtaining a material that acted as a matrix without using any supplementary chemical modifications as it occurs with most of the biobased resins. This material was processed by the conventional transform method (hot compression molding) to form the plates from which the test specimens were extracted. From physicochemical and mechanical characterization, it was found that the resin had obtained a tensile strength of 15 MPa that increased to values of 30 MPa with the addition of 20% of the fibers with alkalization treatment. This behavior indicated a favorable condition of the fiber-matrix interface in the material. Similarly, the evaluation of the moisture adsorption in the components of the composite demonstrated that such adsorption was mainly promoted by the presence of the fibers and had a negative effect on a plasticization phenomenon from humidity that reduced the mechanical properties for all the controlled humidities (47%, 77% and 97%). Finally, due to its physicochemical and mechanical behavior, this new biobased composite is capable of being used in applications such as wood–plastic (WPCs) to replace plastic and/or natural wood products that are widely used today.

## 1. Introduction

Due to the environmental impact caused by the conventional synthetic polymers when they are not properly disposed of at the end of their life cycle, research studies are currently being carried out in the field of polymeric materials that focused on the development of polymers characterized by being biobased, generated from renewable sources such as starches, proteins, hydroxy alkanoates, among others, and by presenting complete biodegradability under composting conditions [1,2,3,4,5,6,7,8]. Unlike traditional synthetic polymers, these materials are not oil-dependent; therefore, they have an added value as a potential alternative to produce eco-friendly materials. Among this family of polymer materials, the natural resins extracted from plants stand out due to the potential use of a wide variety of plants in the ecosystem that constitute a renewable source for polymer obtention [6,7]. These resins are currently being employed for the development of biobased composites, which, in most cases, are used as a partial substitute of reagents on the synthesis of polymers, such as canola oil for the obtention of polyols in order to react with isocyanates for the production of polyurethane adhesives and foams [8], tannin-furfuryl alcohol for thermoset resins [9], soya as polyol for polyurethanes [10], starches, wood, and other natural materials as sources for synthesizing reagents to produce epoxy bio-resins [11], such as modified vegetable oils, sugars, polyphenols, terpenes, colophony, natural rubber, and lignin for chemical synthesis of resins and curing agents for epoxy polymers [12], among others. The Mopa-Mopa resin forms the base of the varnish that is extracted from the *Elaeagia Pastoensis Mora* wild shrub, which belongs to the *Rubiaceae* family and grows in the Department of Putumayo, Amazon region in the Colombian jungle. Twice a year, the plant produces a gelatinous paste, which, through an artisanal process, is transformed into a thin sheet that can be molded to make decorative drawings on pre-painted wood [13]. The resin has been extracted and used by generations of farmers to commercialize it as a raw material mainly for manufacturing and/or restoring handicrafts [14,15]. Although the knowledge of the Mopa-Mopa has been limited to the development of new techniques and products on an artisanal level, there has been a growing interest within the scientific community to analyze and expand the understanding of this polymeric material through research about its physical, mechanical, thermal and chemical properties. Among those studies, Insuasty et al. [16] started the chemical characterization of the Mopa-Mopa resin from solubility tests and several spectroscopic techniques and identified that ethanol and methanol were the best solvents for the resin without losing its physical properties, especially its elasticity. Other studies [17,18] have shown both some physicochemical and thermal properties of the Mopa-Mopa resin and the effect of add polycaprolactone on the properties of binary mixtures using Differential Scanning Calorimetry (DSC), X-ray Diffraction (XRD), Thermogravimetry Analysis (TGA), and Fourier Transform Infrared Spectroscopy (FTIR) tests as well as in determining the semi-crystalline nature of the resin.

Considering the aforementioned aspects, this project developed an efficient extraction process of Mopa-Mopa resin to be implemented as a fully biobased matrix for the production of a biobased composite reinforced with short fibers of fique (25 mm); fique fibers are available in Southwestern Colombia, they are mainly used in packaging and cordage [19]. It is also noted that, due to their good mechanical properties and the technological development associated with its extraction process, fique fibers have also been considered as reinforcement in plastic matrix composite materials. [20,21,22,23]. In the same manner, this work contemplated the obtained biobased composite of Mopa-Mopa resin with 10 and 20% (m/m) of fique fibers, the evaluation effect of the fiber superficial modification process (by alkalization) and the exposure of the material to three different relative humidities (47, 77 and 97%), in its physicochemical and mechanical properties. Furthermore, the new material developed was characterized because it can be processed through conventional transformation methods and because its behavior means that it can also can be used in other applications, such as wood–plastic.

## 2. Materials

The Mopa-Mopa resin used in this research was extracted from the buds of the *Elaeagia Pastoensis Mora*, a plant native to the Department of Putumayo (Mocoa, Colombia) (typical taxonomic classification is provided in Table 1). Furthermore, the obtained resin showed some physical characteristics, such as *ρ* = 1.108 g/cm^3^, T_m_ = 117 °C, and T_g_ = 34 °C. The fique fiber used belongs to the *Furcraea* genus of the *Uña de Águila* (white variety plant), which was provided by the Empaques del Cauca Company located in the city of Popayán, Colombia. The raw material was used to make a short reinforcement with an average length of 25 mm randomly located, and with and without an alkaline treatment inside a Mopa-Mopa resin matrix. Finally, sodium hydroxide used in the alkaline treatment of the fibers and ethanol employed in obtaining Mopa-Mopa resin [24] were reactive grade acquired by the company Técnica Química S.A. (Cali, Colombia).

## 3. Experimental Procedure

### 3.1. Obtaining the Mopa-Mopa Resin

**Figure 1:**
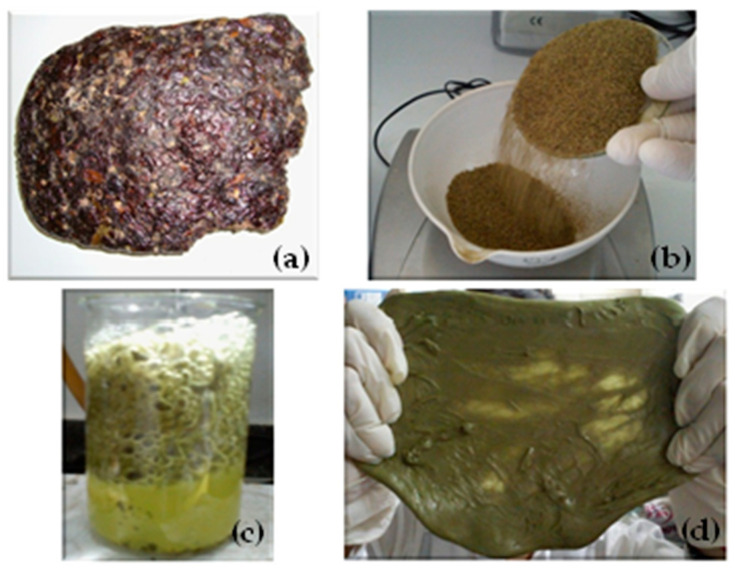
Mopa-Mopa resin: (**a**) in the raw state; (**b**) after the grinding process; (**c**) during the separation process; (**d**) after the extraction process.

Through the use of a manual disk coffee milk, a physical treatment of comminution was carried out to reduce the buds of the Mopa-Mopa tree that were initially agglomerated (Figure 1a), thus achieving an efficient size reduction (Figure 1b). Subsequently, for the obtention processing, 300 mL of a solution of the Mopa-Mopa resin in ethanol was prepared at a concentration of 20% (*m*/*v*) by using a flat-bottom three-necked flask with a capacity of 500 mL and set up in a closed distillation system, keeping the solution under heating at a temperature of 75 °C for 25 min. The remaining fluid was cooled to approximately 40 °C and the solid residues were separated from the mixture, which corresponds to the remains of leaves, seed husks, and stems, among others, by using a vacuum filtration system composed of a porcelain funnel, an Erlenmeyer that received the filtered solution and a vacuum pump Welch model BS-8000 Fisher Scientific (Gardner, MA, USA). This procedure was carried out in two stages, the first stage with absorbent towels, eliminating the larger residues, and the second stage, with qualitative filter paper, to ensure cleaning of the solution. The already filtered mixture was heated again at a temperature between 76 and 78 °C for 45 min, to evaporate as much as possible, that is to say, to concentrate the Mopa-Mopa, taking special care to not degrade the resin; this procedure was conducted in aid of a fractional distillation system to condense the solvent and to regain around 80% of the initial ethanol. Simultaneously to the previous procedure, a volume of 100 to 150 mL of distilled water was heated to a temperature of 100 °C, in which it was added to the Mopa-Mopa concentrated solution. In the making of this mixture, the resin kept suspended on the water surface due to the immiscibility of these two phases (Figure 1c). Subsequently, constant agitation was maintained using a spatula lab until the resin precipitates and can be separated from the liquid. This step corresponded to the 20 min after mixing with distilled water. Similarly, it is important to specify that in this step the remaining solvent that was left in the previous stage is evaporated. The Mopa-Mopa resin obtained was deposited in a watch glass, where it was left to cool for 5 min until reaching a temperature of 25 °C to register the mass. (Figure 1d). A two-stage size reduction process was necessary to carry out the elaboration of test specimens. In a first stage, the material was crushed in a low-speed granulator of the SG-16/20 series until obtaining a particle size of 3 mm, in a second stage, using a manual coffee grinder, until reaching a particle size between 0.3 and 0.5 mm. It is important to note that this resin Mopa-Mopa is characterized by containing a variety of metabolites, including alkaloids, flavonoids, and cardiotonic aglycones [16]. The test specimens were formed in a Carver press model MH 4389-4021 (Wabash, IN, USA) equipped with heating plates and a forced circulation water system) using a pressure of 25,000 lb, and a temperature of 165 °C for 30 min; after that, the obtained plates were die-cut following the shape of the Type IV probes in accordance with ASTM D 638 [25].

### 3.2. Surface Modification by Alkalization of Fique Fibers

Following a similar methodology to that proposed by Valadez et al. [26] for henequen fibers, the alkalization of the fique fibers consisted of pre-drying the material at 100 °C for 24 h, to then carry out a surface treatment by immersing the fibers in an aqueous solution of NaOH (2% *m*/*v*) for 1 h at 25 °C; later, the fibers were washed with distilled water acidified with acetic acid, until reaching a neutral pH, that is, until the washing water had no residual alkaline solution. Finally, the fibers were dried at 60 °C for 24 h.

### 3.3. Preparation of the Biobased Composite

**Figure 2:**
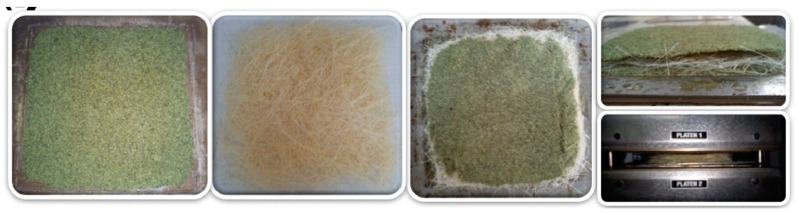
Biobased composite preparation: (**a**) Mopa-Mopa compacted sheet; (**b**) nonwoven fique mats; (**c**) Mopa-Mopa/nonwoven fique mats/Mopa-Mopa laminate; (**d**) molding the biobased composite.

**Figure 3:**
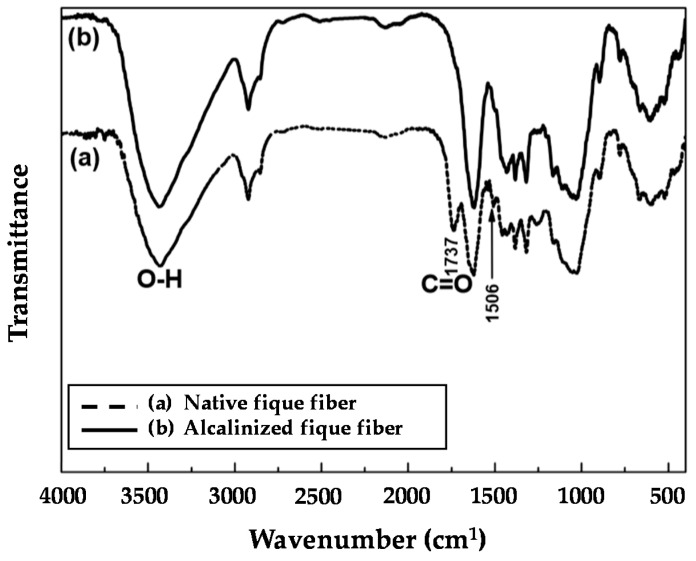
Fourier Transform Infrared Spectroscopy (FTIR) for the fique fibers: (**a**) native; (**b**) alkalized.

For the elaboration of the biobased composite, the components, the Mopa-Mopa resin and the native and alkaline fique fibers, were pressed separately and 25,000 lb of pressure was applied, forming nonwoven fique mats (Figure 2a and Figure 3b). Finally, the components were molded using a sandwich-type layout (Mopa-Mopa sheet–fiber mat–Mopa-Mopa sheet) at a temperature of 170 °C, under the following pressure scheme: 10,000 lb for 3 min, 20,000 lb for 3 min and 25,000 lb for 30 min (Figure 3c,d); reinforcing proportions of 10 and 20% concerning the total mass were maintained.

### 3.4. Fourier Transform Infrared Spectroscopy (FTIR)

For the analysis of the materials, a Fourier Transform Infrared Spectrum 100 model was used. In the case of fique fibers with and without alkaline treatment, the Attenuated Total Reflectance (ATR) technique was followed, working at 100 sweeps and a resolution of 2 cm^−1^. For the Mopa-Mopa resin, the infrared analysis was performed on conditioned specimens at three relative humidity ranges (47, 77 and 97%), as well as on an additional sample that was dried in an oven at 60 °C for 2 h. The Diffuse Reflectance (DRIFT) technique was used in these materials, working at 200 scans and a resolution of 2 cm^−1^.

### 3.5. Moisture Adsorption

Salts of potassium carbonate, sodium chloride, and distilled water were added to desiccators for maintaining constant the relative humidity at 47%, 77%, 97%, respectively, in accordance with ASTM E 104 [27]. Humidity meters were placed in the desiccators to monitor such relative humidity. For the evaluation of the fique fibers, three bundles of the fibers were cut, with masses between 3 and 6 g, both for the native and those that had the alkaline treatment and for each relative humidity. Subsequently, the samples were dried in an oven at 100 °C for 24 h and placed in the respective desiccators, registering their mass. In the case of the Mopa-Mopa resin and the biobased composite, three type IV test specimens were prepared according to ASTM D 638 [25] for each relative humidity and each condition of the composite (proportions of 10 and 20%, with and without treatment alkaline), to then analyze the effect of relativity humidity and exposure time on mechanical properties by applying a tensile test. The data of the mass gain as a function of time (Mt) were taken in each case and the percentage of moisture adsorption (H) was determined based on the model presented in Equation (1), considering the mass after drying in the oven (Ms).

(1) $$H=\left(\frac{\mathrm{Mt}-\mathrm{Ms}}{\mathrm{Ms}}\right)x100$$

### 3.6. Density Determination

This test was carried out on a Mettler Toledo AG245 equipment with a maximum capacity of 210 g and a deviation of 0.001 g. The density value for the Mopa-Mopa resin and the biobased composite in all the study conditions (proportions of 10 and 20% with native and alkalinized fiber) was determined by method A of the ASTM D 792 [28]. In the case of fique fibers with and without alkaline treatment, the density was estimated following method B of the same standard. The theoretical density was estimated with the aid of the rule of mixture model shown in Equation (2)

(2) $$\rho_{b}=\rho_{f}\nu_{f}+\rho_{m}\left(1-\nu_{f}\right)$$

where *ρ* is the density, and the subindices *b*, *f*, and *m* refer to the biobased composite, the fiber, and the matrix, respectively; *ν_f_* is the volume fraction of fiber incorporated into the biobased composite.

### 3.7. Tension Test

The determination of the tensile mechanical properties of the fique fibers with and without alkaline treatment, the Mopa-Mopa resin, and the biobased composite was carried out by a Tinius Olsen model H50KS universal testing machine. An experimental setup was used in the fique fibers, which consisted of the fixing of filaments in cardboard frames, allowing for a better grip of these to the clamp, in accordance to the ASTM D 3822 [29] standard, with a clamp displacement rate of 3 mm/min and taking into account a previous analysis of the distribution of average diameters in each fiber tested. A sample of 140 fibers (70 native and 70 alkalized) was selected at random and estimating about 8 diameter values set for each of the filaments, which generated a standard deviation of 0.03 for the native fibers and 0.02 for the alkalized fibers. For Mopa-Mopa resin and biobased composite, ASTM D638 [25] was followed with type IV specimens and a speed displacement rate of 5 mm/min.

### 3.8. Scanning Electron Microscopy (SEM)

The morphological characterization of the fique fibers, the Mopa-Mopa resin, and the biobased composite were carried out by a Electron Microscope model JMS 6490 LV (Jeol, Mexico D.F., Mexico), in the secondary electrons mode (SEM) and under an accelerating voltage of 20 kV. The chemical microanalysis was performed on several inspection areas using an Energy Dispersive X-ray Spectroscopy (EDS) model IncaPentaFETx3 (Oxford Instrument, Belfast, UK). This technique allowed us to qualitatively know the surface effect of the treatment of the fique fibers, the ductile nature of the Mopa-Mopa resin, and the interface of the biobased composite. The samples were previously bonded on a carbon tape and then metalized with a gold layer between 10 and 50 nm (thin film deposition equipment Model Desk IV at a pressure of 50 m Torr and a time of 60 s, (Denton Vacuum, Moorestown, NJ, USA) in order to generate a conductive surface, analyzing the cross-section of the resin, the biobased composite and the contour of the fique fibers.

## 4. Results and Discussion

### 4.1. Fourier Transform Infrared Spectroscopy (FTIR)

The infrared spectroscopy test of the Mopa-Mopa resin was carried out carefully on four conditioned samples: one dried sample in the oven at 60 °C for 2 h (Reference sample), the remaining samples, at three relative humidities of 47, 77 and 97%. The purpose of this procedure was to learn the effect of water adsorption on the molecular interactions of the resin. Consequently, the following bands for the dry sample could be observed: an intense signal in the region below 3000 cm^−1^, with two bands at 2979.6 and 2951.0 cm^−1^ assignable to the vibration in tension (asymmetric and symmetrical) of the CH bond; at 1751.0 cm^−1^, an intense signal corresponding to the vibration to a tension of the carbonyl group C=O; at 1656.6 and 1474.9 cm^−1^, associated with the vibration in tension C=C, between 1400 and 1000 cm^−1^, other slightly widened bands were observed, most likely related to CO bonds and the deformation vibration of CH bonds. The results found were similar to those reported for the Mopa-Mopa resin in other studies [17,18]. On the other hand, the samples conditioned at 97%, 77%, and 47% relative humidity presented a new band associated with interactions of the hydroxyl group with water molecules at 3284.3 cm^−1^, but with a difference in the peak’s intensity seen in the axis of ordinates that varied in values of 0.017, 0.009 and 0.007, respectively, being greater for the higher humidities. This fact can be correlated with a possible plasticization of the Mopa-Mopa by the influence of the humidity. This phenomenon is similar to the one that happens in the thermoplastic starch [30], whereby incorporating a plasticizer to the cassava starch means that the interactions of the hydroxyl groups are modified within the material and new second-order intermolecular associations (hydrogen bonds) are established with fewer steric hindrances. Table 2 shows the spectral bands for each sample used with the characteristic link type.

Figure 3 shows the infrared spectra obtained in the native and alkalized fibers; in these spectra, the representative bonds of the fundamental constituents of the fiber, such as cellulose, lignin, and hemicellulose, stood out. With the treatment of the fibers in the alkaline solution, it was observed that the peak corresponding to the tension stretch of the carbonyl C=O, which occurred at a wavenumber of 1736 cm^−1^, disappeared from the spectrum, this peak is associated with Ester-type bonds that usually appear in the hemicellulose structure; therefore, the loss of this band indicated that, at least at the surface level, the removal of this component in the fiber was generated. Similarly, it showed a decrease in peaks at 1505 and 2862 cm^−1^ related to the aromatic skeleton and stretching of the -OCH_3_ bond of lignin, respectively, indicating that the lignin surface concentration decreased with alkaline treatment. Similar results were reported by Mina [31] who additionally included the chemical composition for the fique and other natural fibers, finding that the percentage related to lignin and cellulose in the fique was under 14 and 61.2%, respectively.

### 4.2. Moisture Adsorption

**Figure 4:**
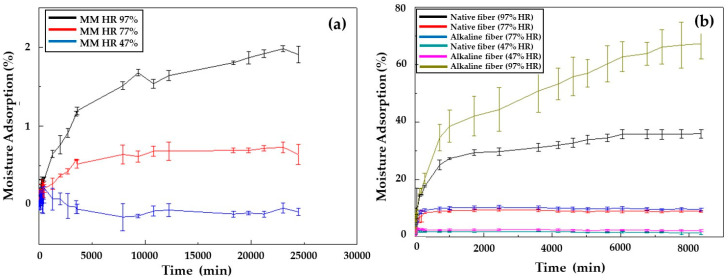
Adsorption isotherms at a relative humidity of 97, 77, and 47% for (**a**) Mopa-Mopa resin; (**b**) native and alkalized fique fibers.

**Figure 5:**
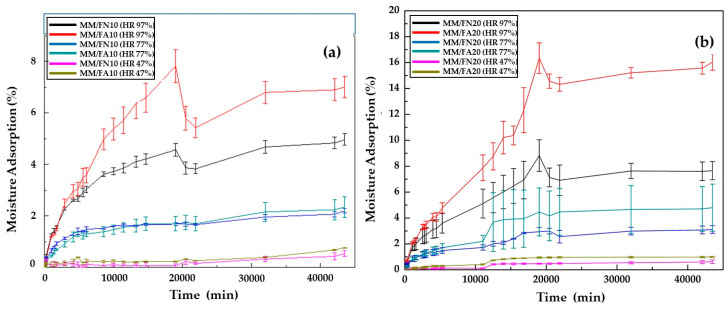
Adsorption isotherms at relative humidities of 97, 77, and 47 for (**a**) biobased composite with 10% native and alkalized fique fibers; (**b**) biobased composite with 20% native and alkalized fique fibers.

This study was carried out considering the conditioning of the material at relative humidities of 47, 77 and 97%, finding in specimens a reduced increase in mass due to low water adsorption in all the different relative humidities controlled. The humidity adsorption values of the equilibrium corresponded to 1.9, 0.64, and 0.09% for the humidity of 97, 77, and 47%, respectively. These data indicated that the Mopa-Mopa resin presented low humidity adsorption compared to other natural polymers, such as thermoplastic starch, that, according to reported results [30], presents about 7% adsorption when reaching equilibrium; for a relative humidity of 43% and 25 °C, these conditions were lower than those used in the present study and with very high adsorption results that were not reached by the Mopa-Mopa resin even at the highest relative humidity studied (97%). The general behavior of the humidity adsorption of the Mopa-Mopa resin in the different atmospheres is shown in Figure 4a, where it becomes evident both the relationship of proportionality between the relative humidity to which the resin was exposed and its adsorption percentage for the humidity of 97 and 77%. Moreover, it must be considered that, during the initial times, the adsorption of the resin occurred at a faster rate (first stage of the curve), followed by an almost constant behavior defined as balance. In a particular case, the curve corresponding to the humidity of 47% showed a decrease in the humidity adsorption because the specimens were not completely dry before being introduced into the desiccator since the resin is very vulnerable to oxidation in prolonged healing periods; therefore, when these presented a higher humidity than the exposure medium, the salts generate a drying effect to balance their humidity, represented in the decreasing behavior of the curve. Fique fibers’ ability to absorb moisture could be influenced by the superficial modification generated by the alkalization treatment, since, with it, some hydrophobic groups are removed from the fiber, such as waxes and pectins, generating a concentration of cellulose on its surface that has hydroxyl groups and a great affinity with water, forming hydrogen bonds and thus increasing its adsorption moisture. This behavior was reflected in the increase in the mass of the fibers when they were exposed to different relative humidities (97, 77, and 47%), with higher adsorption at higher humidities compared to native fibers. Figure 4b shows the curves corresponding to the adsorption isotherms of native fique fibers and of the alkalized fibers, conditioned to the three relative humidities of 47, 77 and 97%. Due to the type of bonds it has, Mopa-Mopa is a material with the proper functionality to form hydrogen bonds with water molecules present in the humidity of the environment, behavior that is similar to that of the fique fibers used as reinforcement and that turn to the increase in the level of moisture adsorption of the Mopa-Mopa/Fique biobased composite concerning the individual behavior of the components, as can be seen in Figure 5a,b. The increase in mass due to the adsorbed water is directly affected by the content of the fibers used, being greater in those with 20% of them. Besides, the alkaline treatment generated a greater absorption of moisture on the part of the biobased composite; this was attributed, as mentioned above, to the fact that the treatment caused an increase in cellulose on the surface of the fiber, which has hydroxyl groups and a great affinity with water, forming hydrogen bonds and increasing their ability to absorb moisture. According to the above, the increase in the mass by water adsorption in the compounds with alkalized fibers could be produced through a diffusion mechanism, due to the presence of micro-spaces between the fiber and the matrix that allowed the water filtration in the material [32]. This phenomenon becomes more important when the amount of fiber increases in the matrix. It is important to mention that, as in the mechanical characterization of the Mopa-Mopa resin, the adsorption of humidity in the same three exposure times was studied in the biobased material, showing a proportional increase in the adsorption of water throughout the exposure time and generating a negative effect on the mechanical performance of the material.

### 4.3. Density Estimation

**Figure 6:**
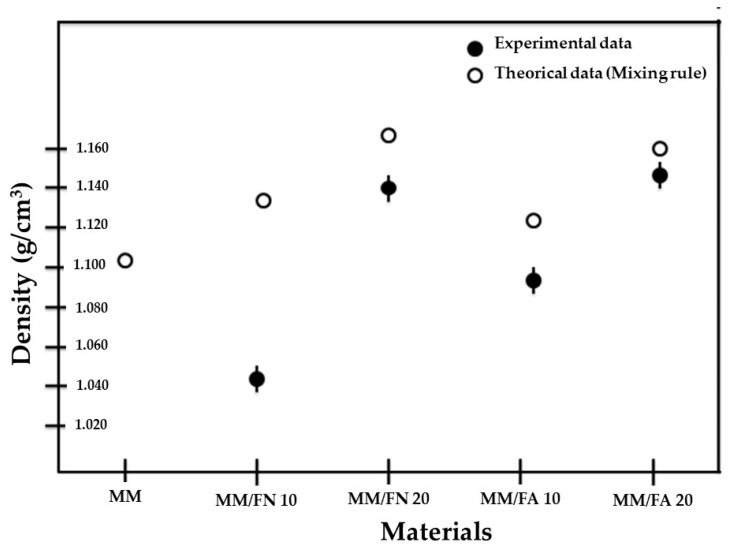
Density data for Mopa-Mopa resin and biobased composite with 10 and 20% native and alkalized fique fibers.

This test was performed on all the manufactured Mopa-Mopa/Fique biobased composite and the Mopa-Mopa resin matrix to establish the effect of fiber incorporation and the surface treatment of the fique on the density of the material, the used values for the theoretical determination of the different compounds were $\rho_{m}$ = 1.108 ± 0.03 g/cm^3^, $\rho_{f}$ = 1.393 ± 0.04 and 1.308 ± 0.06 g/cm^3^ for the native and alkalize fibers, respectively. Figure 6 shows the results obtained and that was compared with the estimated values employing the rule of mixture in Equation (2). By incorporating 10% of fique fibers with and without alkaline treatment, it was found that the density of the composite decreased concerning the density of the matrix. However, the density of the composite with the alkalized fibers was higher than the one with the native fibers. This positive resulting effect from the treatment of the fique fibers in the density of the composite is due to the reduction of voids and/or cavities between the fiber and the matrix that also affects the improvement of the interfacial zone as will be discussed later with the help of SEM images. On the other hand, in the composite made with 20% of native fique fibers and previously treated with NaOH, it was observed that the density increase regarding that of the Mopa-Mopa resin and the compounds with 10% of the fibers is greater than that of the material with 20% alkalinized fibers, presenting a similar trend to the one estimated from the theoretical values. The materials that presented higher values of density also showed greater resistance in tension because they had a better mechanical anchorage; the relationship between the density, the interfacial zone, and the properties of the compounds has been documented in the literature [33]. It is also important to highlight that the dispersion of data decreased with the increase in the volume of fiber, being closer to the theoretical values than the density of the biobased composite reinforced with alkalized fiber.

### 4.4. Tensile Strength

The specimens used to determine the mechanical properties were tested at three time points (t_1_: before exposure to the desiccator; t_2_: 3 days of exposure; t_3_: 15 days) of conditioning, and a relative humidity of 47, 77 and 97%. Table 3 shows the results of the tensile properties for the natural Mopa-Mopa resin at the different relative humidities studied. Here, it can be seen that the material is characterized by having a tensile strength of 10.42 MPa for the conditioning time 1. This value turns out to be low when compared to some conventional synthetic polymers that show strengths around 22 and 30 MPa, as in the case of hight density polyethylene (HDPE) and polypropylene (PP), respectively [34]. However, it has an interesting behavior when compared to materials such as low density polyethylene (LDPE), for which there had been reported values from 5 MPa [35] and some bio-based polymers such as thermoplastic starch that reaches values between 0.23 and 5.5 MPa [31]. On the other hand, it is important to consider that the mechanical properties can be increased with the use of reinforcing materials such as natural fibers, in this case, those of fique. The mechanical properties of the Mopa-Mopa resin varied with the relative humidity to which it was exposed, mainly due to the phenomena of plasticization by water that the material undergoes and which allowed decreases of 69.78, 62.57 and 39.05% for the relative humidities of 97, 77 and 47%, respectively. Such a phenomenon influenced the secondary interactions that were previously discussed through an infrared analysis that is also a condition that occurs in biobased polymers such as thermoplastic starch, for example [30,31,32,33,34,35,36]. When incorporating the fibers in the matrix based on the Mopa-Mopa resin, an increase in the tensile strength of 43.35% for the composite with native fibers and 53.95% for the composite with alkalized fibers was evident, while in the case of the Young′s modulus of the biobased composite, the increase was 34.87 and 37.88% for the fibers without and with alkaline treatment, respectively. These values were a direct function of the content of incorporated fibers, corroborating that the use of these had a positive impact on the mechanical properties of the material. In return, the alkaline surface treatment on the fibers improved the mechanical behavior of the biobased composite by 24.75 and 38.32% compared to that which was reinforced with 10 and 2% fibers without any treatment (native fibers), respectively, due to the increase in roughness and generation of mechanical anchorage. It is important to highlight that the mechanical properties were negatively affected by factors such as humidity and conditioning time, causing a considerable decrease in tensile strength, as can be seen in the data reported in Table 3. This behavior was attributed to the plasticizing effect due to the humidity that the biobased composite adsorbs due to the hydrophilic character of both the matrix and the reinforcement, as previously mentioned. The best mechanical performance in biobased composite associated with the material that was reinforced with 20% alkalized fibers, for which a tensile strength and Young′s modulus of 32.73 and 2128.36 MPa was obtained, respectively; the above for the first evaluation time that corresponded to the specimens before conditioning.

These results were superior to those reported by Delgado et al. [37], who developed wood–plastic composites from a low density polyethylene (LDPE)/hight impact polystyrene (HIPS) matrix reinforced with natural fibers, reaching values below 6 and 70 MPa for the parameters of interest. Additionally, the results reported by Fajardo et al. [38], who developed a PP matrix reinforced with bamboo fibers, that contained values of 12 and 1400 MPa in a tensile strength and Young′s modulus, respectively. In the case of the biobased composites studied in the present study, the mechanical properties achieved were greater, independent of the humidity and conditioning times employed.

### 4.5. Scanning Electron Microscopy (SEM)

**Figure 7:**
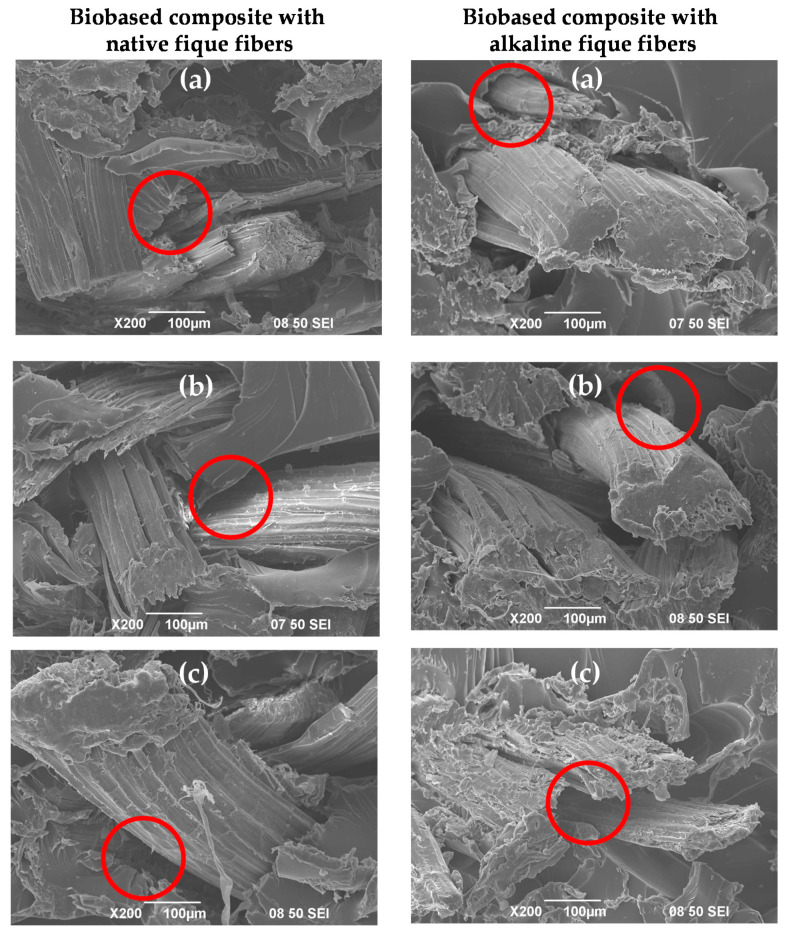
Scanning Electron Images of fracture surfaces of the biobased composite with native and alkaline fibers conditioned at (**a**) a relative humidity of 47%; (**b**) a relative humidity of 77%; (**c**) a relative humidity of 97%. The red circles show voids between the fiber and the matrix.

From SEM micrographs, it was possible to evaluate the quality of the fiber–matrix interface for the biobased composite that was reinforced with the fibers with and without surface treatment. In Figure 7 it can be seen some gaps in the areas located between the matrix and the fiber for the two case studies and that could give an indication of the formation of a weak interface between the phases of the compound. However, thanks to the intrinsic adhesion exhibited by the matrix, the mechanical properties showed increases in the resistance and modulus with the incorporation of the fibers and performing greater values with the fiber alkalized by the mechanical anchorage promoted by the roughness generated by the treatment, as mentioned in other studies [32]. The interface and the mechanical anchorage of the biobased composite are themselves affected by the relative humidity in which the material was exposed (47%, 77% and 97%). It can be seen in Figure 7 that at a higher relative humidity the mechanical anchorage decreases, due to the plasticization of the material caused by the adsorption of water. The lack of adherence between the fibers and the matrix can be seen in the reduction of the amount of matrix on the fibers and the increase in the empty spaces between them (see red circle), being observed a greater intensity at the higher relative humidity. This fact was also visible in the decrease in the mechanical properties of the biobased composites.

## 5. Conclusions

It was possible to develop a new composite material in which the matrix corresponded to the Mopa-Mopa resin, extracted from the bud of the *Elaeagia Pastoensis Mora* plant, characterized by being a fully biobased composite and, unlike most materials reported in the literature, only requiring a physical process of extraction through a closed distillation system that provided a significant yield (92%). In addition, this biobased material could be processed by hot compression molding, which is a conventional transformation process, through which it is possible to manufacture plates of fique fiber-reinforcement and from which test specimens for physical-chemical and mechanical characterizations were produced.

The biobased composite presented a positive synergy between the Mopa-Mopa resin and the fique fibers that were evidenced in the quality of the interfacial zone and from a macro-mechanical perspective, in an increase of the tensile strength and the Young’s modulus as a function of the content of fibers in the material; much more significant increase in those properties was shown when a 20% of fibers superficially modified by an alkalization treatment were incorporated.

On the other hand, it was found that the relative humidity and the conditioning time of the material played an important role in a plasticization phenomenon that generated important reductions in the mechanical properties, especially to 97%, albeit the amount of moisture absorption in the equilibrium was low compared to what was reported for other biocomposites based on natural polymers such as thermoplastic starch. Because of the ease of processing and the physicochemical and mechanical characteristics evaluated that are evident when comparing the results achieved in this new material with those reported LDPE, HIPS and PP biocomposites reinforced with natural fibers, it is possible that the Mopa-Mopa resin/fique fibers biobased composite developed can be used as wood–plastic for the substitution of plastic and/or natural wood, increasing this viability if in further research the effect of integrating additives (lubricants, fillers, pigments, among others) in the composition of the material is studied. 

## Figures and Tables

**Table 1:** Taxonomic classification of the Mopa-Mopa resin.

Description	
**Division**	*Trachelophyta*
**Subdivision**	*Angiospermae*
**Class**	*Dicotyledonae*
**Order**	*Rubiales*
**Family**	*Rubiaceae*
**Genus**	*Elaeagia*
**Species**	*Elaeagia pastoensis Mora*
**Shapes**	*Elaeagia pastoensis Mora fma pastoensis*
	*Elaeagia pastoensis Mora fina acuminata Mora*
**Synonym**	*Elaeagia pastoganomophora*

**Table 2:** Characteristic bands and type of bond for the Mopa-Mopa resin.

Wavenumber (cm^−1^)				Type of Link
Dry Sample	HR 47%	HR 77%	HR 97%	
2979.59	2976.70	2985.38	2977.66	Tension C-H
2951.04	2951.07	2952.45	2974.32	Tension C-H
1751.01	1751.00	1750.81	1751.11	Tension C=O
1656.55	1658.62	1656.55	1654.62	Tension C=C
1474.86	1473.96	1474.21	1475.38	Tension C=C
1394.64	1394.69	1394.80	1394.27	C-O; C-H
1293.28	1293.85	1294.20	1292.83	C-O; C-H
1203.09	1201.79	1203.06	1200.74	C-O; C-H
1123.33	1123.15	1123.70	1123.29	C-O; C-H
1055.42	1056.63	1054.87	1055.59	C-O; C-H

**Table 3:** Tension mechanical properties of Mopa-Mopa resin and biobased composites with native and alkalized fique fibers at 10 and 20% incorporation.

	Time 1 (Before Conditioning)			Time 2 (3 Days of Conditioning)			Time 3 (15 Days of Conditioning)		
	Tensile Strength (MPa)	Strain (mm/mm)	Young’s Modulus (MPa)	Tensile Strength (MPa)	Strain (mm/mm)	Young’s Modulus (MPa)	Tensile Strength (MPa)	Strain (mm/mm)	Young’s Modulus (MPa)
**MM** **HR 97%**	10.42 ± 1.83	1.44 ± 0.63	35.78 ± 5.22	3.15 ± 0.65	1.36 ± 0.35	3.27	2.36 ± 0.82	0.89 ± 0.30	3.18
**MM** **HR 77%**				5.51 ± 1.00	1.37 ± 0.37	11.44	3.9 ± 1.43	1.11 ± 0.36	7.10
**MM** **HR 47%**				7.21 ± 1.59	1.43 ± 0.39	23.03	11.6 ± 2.53	0.89 ± 0.34	20.09
**MM/FN 10** **(HR 97%)**	11.34 ± 4.44	0.09 ± 0.02	739.8	7.21 ± 2.15	0.06 ± 0.03	627.21	6.36 ± 1.47	0.09 ± 0.03	267.04
**MM/FN 10** **(HR 77%)**				10.51 ± 1.92	0.13 ± 0.03	512.84	8.53 ± 1.34	0.14 ± 0.03	325.83
**MM/FN 10** **(HR 47%)**				15.13 ± 1.77	0.07 ± 0.02	920.29	12.16 ± 1.08	0.19 ± 0.04	857.65
**MM/FA 10** **(HR 97%)**	15.07 ± 2.68	0.01 ± 0.02	1321.98	6.73 ± 1.04	0.13 ± 0.04	545.12	4.25 ± 0.68	0.17 ± 0.04	143.37
**MM/FA 10** **(HR 77%)**				11.7 ± 1.34	0.10 ± 0.03	790.57	8.05 ± 0.87	0.14 ± 0.06	325.14
**MM/FA 10** **(HR 47%)**				19.57 ± 2.25	0.1 ± 0.04	817.75	10.2 ± 1.24	0.14 ± 0.03	590.32
**MM/FN 20** **(HR 97%)**	20.02 ± 7.99	0.05 ± 0.01	1135.86	7.52 ± 1.55	0.11 ± 0.03	288.54	6.09 ± 0.94	0.25 ± 0.06	201.62
**MM/FN 20** **(HR 77%)**				10.96 ± 2.25	0.09 ± 0.02	688.83	10.87 ± 2.56	0.04 ± 0.02	621.69
**MM/FN 20** **(HR 47%)**				16.17 ± 4.55	0.04 ± 0.01	1022.74	15.22 ± 2.54	0.02 ± 0.01	872.45
**MM/FA 20** **(HR 97%)**	32.73 ± 10.13	0.02 ± 0.01	2128.36	12.85 ± 2.48	0.08 ± 0.03	483.14	5.98 ± 0.74	0.12 ± 0.04	388.62
**MM/FA 20** **(HR 77 %)**				16.65 ± 4.14	0.06 ± 0.02	1667.61	7.43 ± 2.76	0.09 ± 0.02	400.55
**MM/FA 20** **(HR 47 %)**				28.85 ± 6.63	0.05 ± 0.01	1667.61	18.49 ± 4.23	0.03 ± 0.01	1417.07
